# The value of phosphohistone H3 as a proliferation marker for evaluating invasive breast cancers: A comparative study with Ki67

**DOI:** 10.18632/oncotarget.17775

**Published:** 2017-05-10

**Authors:** Ji-Ye Kim, Hyang Sook Jeong, Taek Chung, Moonsik Kim, Ji Hee Lee, Woo Hee Jung, Ja Seung Koo

**Affiliations:** ^1^ Department of Pathology and Translational Genomics, Sungkyunkwan University School of Medicine, Samsung Medical Center, Seoul, Republic of Korea; ^2^ Department of Pathology, Yonsei University College of Medicine, Severance Hospital, Seoul, Republic of Korea

**Keywords:** PHH3, invasive breast cancer, proliferation, reproducibility, prognostic value

## Abstract

**Background:**

Established measurements of proliferation in breast cancer are Ki67 and mitotic-activity-index (MAI), with problems in reproducibility and prognostic accuracy. Phosphohistone H3 (PHH3), a relatively novel IHC marker is specific for mitosis with good reproducibility. We hypothesized that PHH3 would be more reproducible and better represent proliferation than Ki67.

**Results:**

PHH3 identified easily-missed mitosis by MAI, as demonstrated by upgrading M grade at diagnosis (*n =* 29/218, evenly distributed). PHH3 accurately found hot-spots, supported by mitotic count agreement between low-power and 10HPFs (R^2^ = 0.999; *P* = 0.001). PHH3 was more reproducible than Ki67, measured by five-rater inter-class correlation coefficient (0.904 > 0.712; *P =* 0.008). Finally, despite a relatively short follow-up (median 46 months; 7 recurrences) PHH3 was the only variable correlated with disease-free survival (*P =* 0.043), while all other conventional clinicopathologic variables, including Ki67 (*P =* 0.356), did not.

**Materials and Methods:**

We compared Ki67 and PHH3 for 218 breast cancer surgical cases diagnosed from 2012 to 2013 at Severance hospital. The most representative invasive breast cancer surgical slides were immunohistochemically stained for Ki67 and PHH3.

**Conclusions:**

Poor reproducibility and inadequate representation of proliferation of Ki67 and MAI may be improved by PHH3, allowing better accuracy in breast cancer diagnostics.

## INTRODUCTION

Of all human malignancies, breast cancer is notorious for heterogeneity, thus difficult to predict malignant behavior [1]. Therefore measuring proliferation is important to classify and predict biologic behaviors of breast cancers. The two best-known methods of measuring proliferation are, Ki67 and mitotic activity index (MAI). MAI is included in the standard breast cancer pathology report as part of the histologic grade and considered the most important component to predict prognosis [2]. Ki67 is commonly used in many laboratories to distinguish those with significant risk of relapse to warrant adjuvant cytotoxic chemotherapy and is a criterion in breast cancer subtyping [3, 4]. However, despite these important roles, Ki67 and MAI both have notable weaknesses in reproducibility, which is problematic in diagnostics.

In terms of a reliable, reproducible marker for diagnostics, Ki67 is particularly problematic in heterogeneous tumors as breast cancer, causing disagreement in field selection for assessment. In addition, depending on the standards of each rater, Ki67 may be considered positive for a wide range of stain intensities, culminating in poor reproducibility [5]. Another notable weakness of Ki67 is its adequacy in representing proliferation. Ki67 is a DNA-binding protein expressed in all active phases of the cell cycle (G1, S, and G2 phases). Despite universal acceptance of Ki67 as a proliferative marker, several studies have disapproved, particularly because cells in the G1 phase have shown uncertain destinies [6–8].

While MAI is deemed best amongst available proliferative markers for reflecting proliferative potential in the College of American Pathologists’ consensus statement in 1999 and the Union for International Cancer Control [9, 10], its reproducibility was consistently reported unsatisfactory. Factors contributing to low reproducibility may be from difficulties in discriminating mitotically active areas in Hematoxylin and Eosin (H&E) as well as coexistence of cells that mimic mitosis, such as hyperchromatic, karyorrhectic, or apoptotic cells, which leads to low reproducibility even among trained pathologists [11].

Such problems in conventional proliferative markers may be solved by the use of phosphohistone H3 (PHH3). Histone H3 is a nuclear core histone protein of DNA chromatin, with an important role in chromosome condensation and cell-cycle progression during mitosis and meiosis after phosphorylation of serine-10 and serine-28 residues. Phosphorylation occurs during late G2 to early prophase, while dephosphorylation occurs slowly from late anaphase to early telophase. Therefore in metaphase, histone H3 is always heavily phosphorylated and positive for PHH3, whereas interphase does not or minimally express PHH3 – a property that allows PHH3 to stain only mitotically active cells, therefore proliferation-specific [9].

PHH3 has been verified in multiple studies concerning various tumors (colorectal adenocarcinoma, ovarian serous adenocarcinoma, pulmonary neuroendocrine carcinoma, uterine smooth muscle tumors, astrocytomas, and meningiomas), for its sensitive and specific role as a marker of mitotic figures (MFs) and excellent correlation with outcome [12–17]. In breast cancer, MAI was also strongly correlated with PHH3 [18]. In that study, the authors proposed that PHH3 had potential to assist in breast cancer grading because PHH3 more accurately detects MFs than traditional MAI. Supported by these reasons, we hypothesized that PHH3 may be superior to the existing marker Ki67 in terms of reproducibility and better represent proliferation.

In a similar study by Gerring et al. [19] reported that PHH3 was a stronger predictor of survival at 5 years after diagnosis than Ki67 (hazard ratio 4.35 > 2.44) and better separated risk of death in patients aged >45 years. However, the study used Tissue Microarrays (TMAs), which is not representative of tumor heterogeneity.

This comparative exploratory study improves on previous studies because it was conducted on more than 200 cases of surgical slides of the most representative tumor section, simulating the actual setting of breast cancer diagnosis. In addition, we address all problems associated with conventional markers of proliferation (MAI and Ki67) and compare it to the more novel marker, PHH3.

## RESULTS

### Patient characteristics

This study examined 218 consecutive primary breast cancer (stage I∼III) cases from Jan. 2012 to Dec. 2013 at Severance hospital, Seoul, Korea. Age of patients ranged from 26 to 83 years (mean 53.8) and mean tumor diameter was 1.87 cm (range 1.0–9.0). Three-fourths (76.6%) had pathologically negative nodes (Table 1). estrogen receptor (ER) and progesterone receptor (PR)positive rates were 78.0 and 51.8%, respectively; positive rate of HER2 cases was 15.1%. Distribution of patients according to breast cancer subtypes were as follows; Luminal A (105 cases), Luminal B (65 cases), HER2 enriched (36 cases), and triple negative (12 cases). The median observation period was 46 months.

**Table 1:** Association between clinicopathologic characteristics and Ki-67, PHH3 expressions

Parameters	Total *N* = 218 (%)	Ki-67 LI (cut-off: 43.0%)			PHH3 (cut-off: 0.30%)		
		Low gr. (%)	High gr. (%)	*P*	Low gr. (%)	High gr. (%)	*P*
Age (years)				0.477			0.984
≤ 50	88 (40.4)	75 (34.4)	13 (6.0)		52 (23.9)	36 (16.5)	
> 50	130 (59.6)	106 (48.6)	24 (11.0)		77 (35.3)	53 (24.3)	
Nuclear grade				0.076			0.392
1/2	161 (73.9)	138 (63.3)	23 (10.6)		98 (45.0)	63 (28.9)	
3	57 (26.1)	43 (19.7)	14 (6.4)		31 (14.2)	26 (11.9)	
Mitotic grade				0.112			0.160
1	137 (62.8)	118 (54.1)	19 (8.7)		86 (39.4)	51 (23.4)	
2/3	81 (37.2)	63 (28.9)	18 (8.3)		43 (19.7)	38 (17.4)	
Histologic grade				0.060			0.363
I	62 (28.4)	52 (23.9)	10 (4.6)		40 (18.3)	22 (10.1)	
II	100 (45.9)	88 (40.4)	12 (5.5)		60 (27.5)	40 (18.3)	
III	56 (25.7)	41 (18.8)	15 (6.9)		29 (13.3)	27 (12.4)	
Tumor stage				0.150			0.960
T1	149 (68.3)	120 (55.0)	29 (13.3)		88 (40.4)	61 (28.0)	
T2/T3	69 (31.7)	61 (28.0)	8 (3.7)		41 (18.8)	28 (12.8)	
Nodal metastasis				0.883			0.789
Absent	167 (76.6)	139 (63.8)	28 (12.8)		98 (45.0)	69 (31.7)	
Present	51 (23.4)	42 (19.3)	9 (4.1)		31 (14.2)	20 (9.2)	
Estrogen receptor				0.093			0.258
Negative	48 (22.0)	36 (16.5)	12 (5.5)		25 (11.5)	23 (10.6)	
Positive	170 (78.0)	145 (66.5)	25 (11.5)		104 (47.7)	66 (30.3)	
Progesterone receptor				0.431			0.388
Negative	105 (48.2)	85 (39.0)	20 (9.2)		59 (27.1)	46 (21.1)	
Positive	113 (51.8)	96 (44.0)	17 (7.8)		70 (32.1)	43 (19.7)	
HER-2 status				0.481			0.175
Negative	185 (84.9)	155 (71.1)	30 (13.8)		113 (51.8)	72 (33.0)	
Positive	33 (15.1)	26 (11.9)	7 (3.2)		16 (7.3)	17 (7.8)	
Molecular subtype				0.355			0.192
Luminal A	105 (48.2)	88 (40.4)	17 (7.8)		70 (32.1)	35 (16.1)	
Luminal B	65 (29.8)	57 (26.1)	8 (3.7)		34 (15.6)	31 (14.2)	
HER-2	36 (16.5)	27 (12.4)	9 (4.1)		19 (8.7)	17 (7.8)	
TNBC	12 (5.5)	9 (4.1)	3 (1.4)		6 (2.8)	6 (2.8)	

### Expression patterns: Ki67 and PHH3

For each case Ki67 was scored as recommended by the International Ki67 in Breast Cancer Working Group [20]; four HPFs (objective 40x) that best represented the overall tumor were selected from the invasive front. The exact same fields of Ki67 were appropriately marked for PHH3 (Supplementary Figure 1). Both markers were scored by the average percentage of positive cells in those four fields.

**Figure 1:**
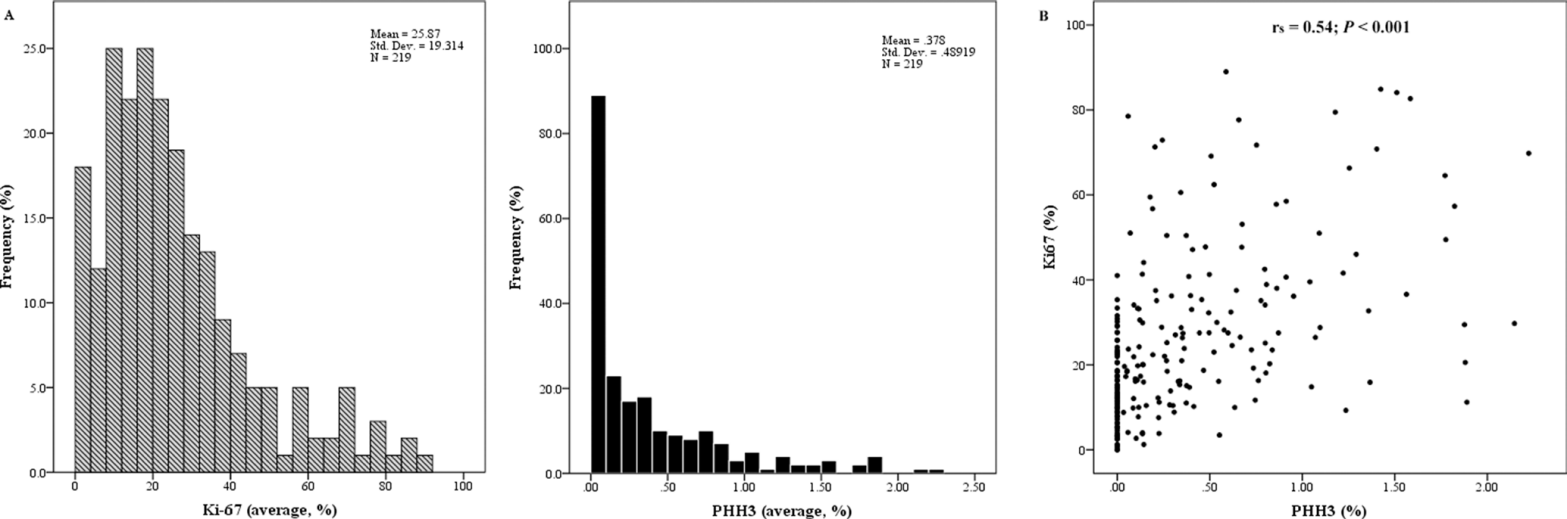
Moderately positive linear correlation between Ki67 and PHH3 Ki67 and PHH3 both had left-skewed distribution of data (**A**). Linear regression coefficient (R^2^) was 0.147 (*P* < 0.001, **B**).

There was an overall tendency for Ki67 and PHH3 to be positively correlated. Histogram and Shapiro–Wilk test (*P* < 0.001) revealed a left-skewed distribution of both markers (Figure 1A). Spearman correlation was 0.54 (*P* < 0.001), demonstrating a moderately positive linear relationship between the two (Figure 1B).

**Figure 2:**
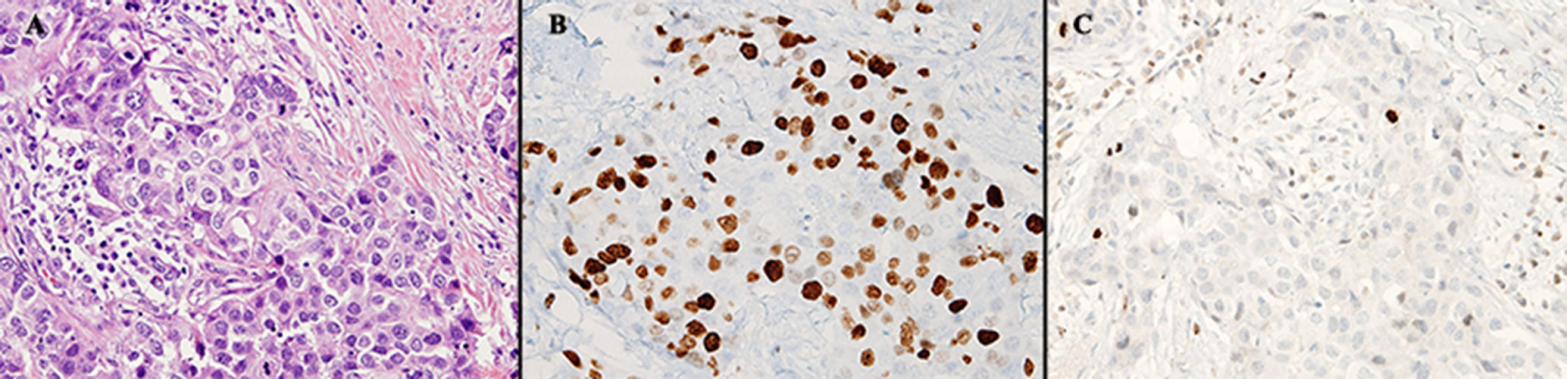
Comparison of same tumor area in H&E, Ki67 (IHC) and PHH3 (IHC) Three mitotic figures were noted by H&E (**A**) amongst apoptotic, necrotic cells - common mimickers of mitosis. Ki67 stained in various intensities (**B**) while the same area for PHH3 stained a few, specific to tumor cells undergoing mitosis (**C**). Images were taken at 40× objective.

Overall, PHH3 was expressed in significantly fewer cells than Ki67 (mean: 0.393 ± 0.568% < 36.67 ± 26.08%, *P* < 0.001). Ki67 had a wide range (min. 0%, max. 89.00%; IQR1 11.66%, IQR3 34.09%) while range of PHH3 was much narrow (min. 0%, max. 2.22%; IQR1 0%, IQR3 0.59%). Ki67 had wide range of intensities and more frequently expressed in non-tumor cells, whereas only a few cells expressed PHH3, usually with strong intensities (Figure 2). PHH3 stained cells were reconfirmed by morphology (nuclei containing condensed chromatin) to accurately count mitotic cells.

### Ki67 and PHH3 cut-off values by contal and O’Quigley’s method was chosen

Ki67 and PHH3 cut-off values were chosen by Contal and O’Quigley’s method to select the most appropriate cut-off based on clinical events (recurrences, Supplementary Table 2). Contal and O’Quigely’s method finds the best cutpoint in a continuous variable with regards to a survival outcome and time to event based on the log rank test statistic. The resulting cut-off of Ki67 was 43.0% while PHH3 was 0.03%, by which Ki67 and PHH3 were converted into categorical variables in low and high groups.

Low and high groups of Ki67 and PHH3 were shown to have no significant correlations with traditional clinicopathologic parameters (Table 1).

### PHH3 had near-perfect inter-rater agreement in staining interpretations

To evaluate interpretation of positivity only and remove discrepancy caused by selection of different fields 221 randomly selected photographs taken at HPFs were assessed independently by two trained pathologists of the same institution. In consideration of the continuous nature of values, inter-class correlation coefficient (ICC) measured inter-rater agreement. ICC revealed a near-perfect agreement in both methods (microscope 0.984, 95% confidence interval (CI) 0.970–0.991; photographs 0.953, 95% CI 0.937–0.964), validating PHH3 as a reproducible measure in selection of appropriate fields as well as clear and unequivocal interpretation of positive cells.

### PHH3 had significantly superior inter-rater agreement compared to Ki67, evaluated by five raters

Inter-rater agreement of Ki67 was assessed for 30 Ki67 stained slides on the microscope by five different raters (Kim JY, Jeong HS, Chung T, Kim M, Lee JH), independently. Each rater rated four consecutive fields of the hot-spot area and rated each area for positive cell percentage and the average was recorded. The resulting ICC was 0.712 (95% CI 0.598–0.856), considered good agreement.

Inter-rater agreement of PHH3 was assessed for the same 30 cases as Ki67, independently rated on the microscope by the same five raters. Each rater selected four consecutive fields of the hot-spot area, equal to the method rated for Ki67 and the sum of positive cells was recorded. The resulting ICC was 0.904 (95% CI 0.869–0.960) – excellent agreement.

ICC for both Ki67 and PHH3 were compared using the dependent ICC comparisons test, which compares dissimilar methods for the same set of cases (Supplementary Figure 2). The comparisons test revealed that ICC of PHH3 was significantly higher than that of Ki67 (0.904 > 0.712; *P* = 0.008), demonstrating PHH3’s superior reproducibility to Ki67.

### PHH3 was more sensitive for detecting mitosis than MAI

With the purpose of comparing M grades scored by PHH3 and those by H&E (MAI), we counted MFs as detected on PHH3 stained slides for 10 contiguous HPFs in areas of highest mitotic activity. PHH3-labeled-MFs were easily seen and permitted quick identification of hot-spots. The counted mitotic number was converted to M grade of the Nottingham grading system, as follows; grade 1 for less than or equal to 7 mitoses per 10 HPFs; grade 2 for 8–14 mitoses per 10 HPFs; grade 3 for equal to or greater than 15 mitoses per 10 HPFs. This newly scored M grade of PHH3 was compared to the M grade assessed by MAI of H&E slides.

Based on these comparisons, there were more up-graded than down-graded cases (29 > 17 cases, Table 2) compared to M grade at diagnosis. As a sensitive marker of mitosis, PHH3 tended to reveal mitosis that were overlooked by MAI, which caused up-grading. 17 cases which were downgraded upon PHH3 counting were predominantly from older blocks, suggesting loss of antigen preservation as the cause of down-grading. The up-graded 29 cases for PHH3 M grade were evenly distributed across the years. This is noteworthy because it highlights an inherent problem of the M grade evaluated by H&E (MAI), which may under-grade the proliferative potential of tumor by under-detection of mitosis.

**Table 2:** PHH3-labeled-MFs counted at 10 HPFs compared with M grade at diagnosis

Parameters	Number of cases	Percent (%)
down-graded	16	7.2
up-graded	29	13.2
concordance	175	79.5
Total	220	100

### PHH3 accurately identified mitotic hot spots at low power

**Figure 3:**
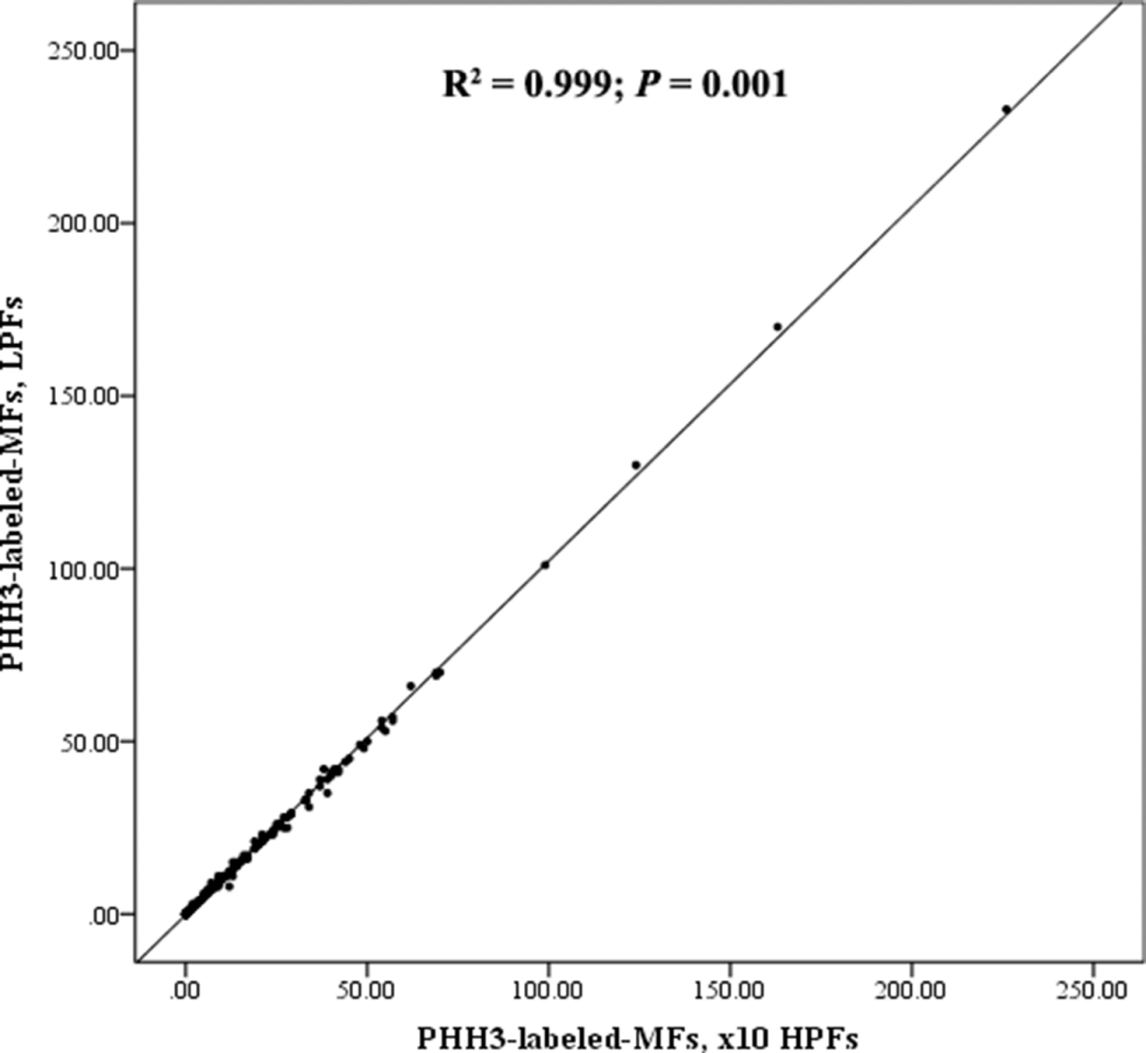
Scatter plot for PHH3-labeled-MFs x10 HPFs and PHH3-labeled-MFs at LPFs There was a strong linear correlation between PHH3-labeled-MFs counted at 10 HPFs and PHH3-labeled-MFs counted at LPFs (four LPFs in approximate area equivalent to 10HPFs); linear regression coefficient R^2^ = 0.999, *P* = 0.001.

To demonstrate efficacy of PHH3 in identifying areas of mitotically active hot spots in heterogeneous tumors, PHH3-labeled-MFs were counted for low-power fields (LPF; objective 10x). Then it was compared with counts from 10 HPFs. Correlation between PHH3-labeled-MFs at 10HPFs and PHH3-labeled-MFs at LPFs was high (Figure 3, R^2^ = 0.999; *P* = 0.001). As there was no change in M grade in both methods, κ statistics showed a perfect fit (κ = 1).

### Amongst conventional clinicopathologic factors, PHH3 was the only factor associated with survival

**Figure 4:**
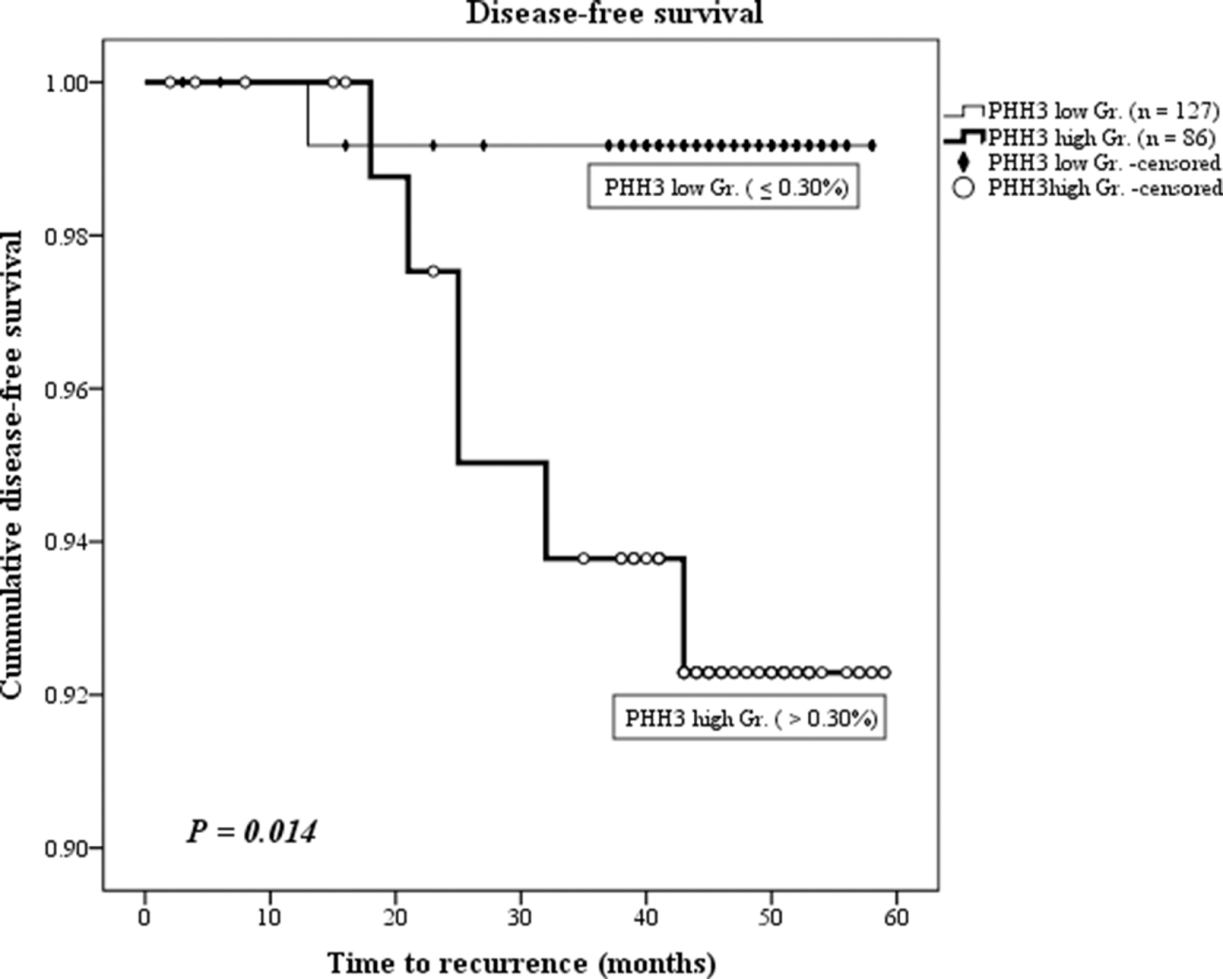
Disease-free survival shown by Kaplan–Meier curve Total of 213 cases (5 cases lost in follow up) were divided into PHH3 low and high groups. High PHH3 expression (> 0.03%) was significantly associated with shorter recurrence-free survival (mean survival time, 56.60 months < 57.63 months).

Median follow-up was 46 months (range 2–73 months) with seven local recurrence/metastases. Ki67 scored for the average of four representative fields, and equally scored PHH3 was analyzed by Cox regression and Log-rank statistics. When PHH3 and Ki67 were handled as continuous variables, there was no significance in Cox regression analysis (Table 3). However when dichotomized by cut-off values generated from Contal and O’quigeley’s method, PHH3 low and high groups was the only factor significantly associated with disease-free survival by Log-rank statistics and Cox regression univariate analysis (*P* = 0.014 and 0.043, respectively; Table 3, Figure 4), despite the short follow-up time; Ki67 and conventional clinicopathologic variables all failed to be prognostically significant. PHH3 low group had a mean survival time of 57.63 months (95% CI 56.90–58.35) which was significantly longer than mean survival time of 56.60 months (95% CI 54.70–58.50) for the PHH3 high group. Hazard ratio was 8.907 (95% CI 1.07–73.99). To compensate for censored events which outnumbered effective events, Harrell’s C-index was calculated, still establishing PHH3’s superior ability for prediction than Ki67 (PHH3 0.723 > Ki67 0.645). The Akaike information criterion (AIC) was also calculated for Ki67 and PHH3 to determine which is more explanatory and informative in predicting survival. The AIC is a commonly used measure for comparison of competing models, and a smaller AIC indicates the preferred model. AIC of PHH3 was smaller than Ki67 (69.453 < 72.607), indicating better predictive ability in survival.

**Table 3:** Recurrence-free statistics of conventional clinicopathologic variables and Ki-67, PHH3

Parameters	Log rank statistics		Cox regression, univariate analysis	
	Recurrence/Total number	*P*	Hazard ratio (95% CI)	*P*
Age (years)		0.388	0.524 (0.117–2.340)	0.397
≤ 50	4/87			
> 50	3/126			0.255
Nuclear grade		0.240	2.386 (0.534–10.664)	
1/2	4/158			
3	3/55			
Mitotic grade		0.663	1.392 (0.311–6.220)	0.665
1	4/134			
2/3	3/79			
Histologic grade		0.451	1.504 (0.543–4.165)	0.433
I	2/61			
II	2/98			
III	3/54			
Tumor stage		0.537	1.594 (0.357–7.125)	0.541
T1	4/146			
T2/T3	3/67			
Nodal metastasis		0.780	1.271 (0.246–6.549)	0.781
Absent	5/163			
Present	2/50			
Estrogen receptor		0.585	0.631 (0.122–3.255)	0.588
Negative	2/46			
Positive	5/167			
Progesterone receptor		0.582	0.659 (0.148–2.946)	0.594
Negative	4/105			
Positive	3/113			
HER-2 status		0.994	0.957 (0.115-7.950)	0.994
Negative	6/181			
Positive	1/32			
Molecular subtype		0.186	1.501 (0.729-3.089)	0.270
Luminal A	1/103			
Luminal B	4/64			
HER-2	2/34			
TNBC	0/12			
Ki-67 (cut-off: 43.0%)		0.343		
Low	5/179		Continuous 1.019 (0.988–1.052)	0.227
High	2/34		Categorical (Low vs. High) 1.365 (0.265–7.035)	0.356
PHH3 (cut-off: 0.30%)		0.014		
Low	1/127		Continuous 1.827 (0.556–5.999)	0.321
High	6/86		Categorical (Low vs. High) 4.826 (1.080–21.567)	0.043

## DISCUSSION

PHH3 was expressed in significantly fewer cells than Ki67, demonstrating better selectivity for a specific phase in the cell cycle. PHH3 identified easily missed mitosis by MAI, causing a tendency for upgrade in M grade of previously diagnosed cases. Between Ki67 and PHH3, PHH3 had significantly better reproducibility, confirmed by ICC. PHH3 also accurately identified mitotically active areas through mitotic count agreement between low power and 10HPFs, indicating easy recognition of mitotic hotspots, which would permit appropriate selection of fields to grade mitosis. Finally, despite a relatively short follow-up (median 46 months) and despite that PHH3 was scored by Ki67 scoring method and not of its own mitotic counts by 10 HPFs, PHH3 was the only significant factor correlated with disease-free survival; conventional clinicopathologic variables, including Ki67, did not correlate with disease-free survival.

Previous studies have noted on the excellence of predictability of PHH3 in long term follow up; all were at least twice the duration of ours – the shortest reported being 85 months and the longest, 168 months (14 years) [19, 21–23]. Skaland et al. studied node-negative invasive breast cancers in patients less than 55 years old treated with adjuvant systemic chemotherapy with follow-up of median 168 months [22]. Gerring et al. compared Ki67 with PHH3 on TMAs and discovered that PHH3 was a stronger predictor of survival than Ki67 with median follow-up of 85 months [19]. For premenopausal node-negative breast cancer patients, PHH3 was also significant for survival with median follow-up of 10.8 years [21]. Our study is notable in that PHH3 demonstrated as a significant marker of survival even in such short term follow up, shortest reported so far.

In this study, direct comparison of PHH3 with the existing marker Ki67 was possible because PHH3 was scored according to Ki67 scoring method; standardized Ki67 scoring of average percentage of positive cells in four representative fields, was equally applied on PHH3. However, it should be noted that in practice, it is unusual to score PHH3 in terms of Ki67. PHH3 is essentially a mitotic marker, usually scored by counting mitosis in 10 HPFs. In this study, for fair comparison PHH3 was scored by the Ki67 scoring method, which does not capture the full potential of PHH3 in its coverage of mitosis. Therefore, in this study, the predictive power of PHH3 is likely to have been undermined, but despite of these limiting conditions, it is noteworthy that it still outperformed Ki67 in survival prediction.

Surprisingly, none of the clinicopathologic variables, including M grade and H grade correlated with Ki67 or PHH3. This finding may prove the more subjective nature of mitotic grading, which also influences histologic grade. Also, because breast cancer prognosis is multifactorial [1], it may not be necessary for PHH3 to correlate with known clinicopathologic variables, which are rarely independently useful to evaluate prognosis of breast cancer. Furthermore, in our study, M grade and H grade did not correlate with neither log rank nor cox regression survival statistics while PHH3 did, verifying that PHH3 is clinically more relevant than the traditional H&E mitotic grading (MAI).

Ki67 and PHH3, both IHC markers, allow convenient distinction between positive and negative cells. Problems of reproducibility of Ki67 concerned interpretational variations in stain intensities as well as selection of fields in heterogeneous breast cancers [24]. In contrast, PHH3 offers a unique feature of reconfirmation of mitosis by morphology (condensed chromatin), allowing better agreement as well as accuracy of detection [14]. In light of this advantage, PHH3 has been reported to closely match with mitotic index [18, 25].

Ki67 use in breast cancer diagnostics has been disputed for inadequate representation of proliferation and poor inter-rater reproducibility. Ki67 may be unsuitable to represent proliferation as it marks all cells in the cell cycle, except for G0 phase. Several studies have shown that G1 phase cells had uncertain destinies, therefore inclusion of G1 would be inaccurate to measure proliferation [6–8]. However, the more widely recognized flaw of Ki67 is low inter-rater reproducibility. Especially problematic in heterogeneous breast cancers, much of the problem was credited to a lack of scoring consensus amongst experts; some advocate selective use of hotspots in assessment of Ki67 while others favor taking average of the invasive front [20]. Yet, even after standardization of the 2011 International Ki67 in Breast Cancer Working Group, which recommended taking the overall average of tumor, including hotspots [5, 20], problems in reproducibility was consistently reported. No single factor (counting method, threshold for positivity, area chosen to score, or staining methodology) explained the cause of low reproducibility of Ki67, according to the International Ki67 Reproducibility Study of 2013 [26]. This multicenter study reported there was substantial variability in inter-laboratory reproducibility demonstrated by moderate ICC with a wide ranged 95% CI (central staining: ICC = 0.71, 95% CI = 0.47 to 0.78; local staining: ICC = 0.59, 95% CI = 0.37 to 0.68), which concluded the clinical utility of Ki67 in breast cancer to be “elusive”. Another study reported high variability among 15 pathologists in assessing three breast carcinoma cases (κ 0.04–0.14) [27]. Ki67’s poor kappa score was contrasted to the excellent kappa score of a different study assessing reproducibility of PHH3 among three pathologists (κ 0.87, 0.79, 0.76) [18].

Our study confirmed those very limiting factors of Ki67 which contributes to low reproducibility. We demonstrated Ki67 staining with wide range in intensities; depending on the rater, the threshold for positive cells would be set differently. In contrast, PHH3 displayed little variations in expression intensities and only counted for strongly stained cells then reconfirmed by morphology, which allows accuracy and consistency amongst raters. Reproducibility was tested by inter-rater agreement test of Ki67 and PHH3 on hot spots, where indeed Ki67 had a significantly lower ICC than PHH3 (0.701 < 0.904; *P* = 0.008).

We also demonstrated PHH3 to have better sensitivity for detecting mitosis than MAI; there was a tendency for up-grading of M grade when re-evaluated by PHH3. LPF assessments also correlated well with HPF enumerations signifying that mitotically active areas could be located in low power, resolving the weakness of MAI, which is impossible to identify mitotically active areas properly for accurate measurement of mitosis. Aside from the current study of breast cancers, meningioma is another tumor where mitosis is important for tumor grading where PHH3 proved to produce rapid, reliable grading [13, 28].

PHH3 targets cells in mitosis, therefore should theoretically match with mitotic counts determined by microscopic analysis of the H&E-stained slide. However, M grade counted by PHH3 did not always match with the M grade counted on H&E (MAI). A possible explanation for the discrepancy is the subjectivity associated with mitotic activity index and inaccurate localizations of mitotically active areas [28–30]. On a similar note, studies noting superior survival correlations of Ki67 compared to mitotic index may be explained by possible masked, unidentified mitosis which may have underestimated the actual proliferative potential of mitotic index [31, 32]. Our study demonstrated the underestimation of mitotic power on H&E (MAI) through upgraded M grades by PHH3. In addition, survival statistics proved M grade insignificant in prognostication, in contrast to PHH3 which was significantly associated with disease-free survival. These findings suggest that the M grade evaluated by conventional H&E microscopic analysis (MAI) may not fully represent a tumor’s proliferative potential. In addition, a recent study demonstrated excellent correlation between disease-free-survival and Nottingham M grade using PHH3 [25]. Supported by these findings, PHH3 may improve M grade accuracy of the Nottingham grading system through better identification of mitosis.

In a comparable study by Gerring et al. [19], PHH3 was also superior to Ki67 in predicting patient survival. However, their study had longer follow-up (median 85 months; maximum 191 months) with more accumulated data for survival analysis (54 deaths out of 108 patients). Despite the advantage of long follow-up, the study tested on TMAs, which does not address the dilemma of tumor heterogeneity in every-day breast cancer diagnostics. Our study was representative of the actual diagnostic practice by using surgical slides and demonstrated meaningful associations with survival in a notably short follow-up period of median 46 months. Taken together, our study provides stronger evidence that PHH3 is a superior prognostic marker to Ki67.

To our knowledge, this is the first study that addresses all problems associated with conventional markers of proliferation (MAI and Ki67) and highlights advantages of PHH3. PHH3 is specific to mitosis and reliably discriminates true proliferating cells from proliferation-mimicking cells, a notable advantage compared to MAI or Ki67. It is particularly valuable in heterogeneous tumors because it accurately identifies mitotically active areas with discrete expressions, permitting good agreement among raters. Our study confirms PHH3’s ability to accurately measure proliferation, through its clinical importance as the only marker amongst known clinicopathologic variables to correlate with survival.

Ki67 and MAI have been the mode of evaluating breast cancers despite flaws in reproducibility for there had been no better alternative. Findings of our study advocate the routine use of PHH3 in diagnostics to reinforce weaknesses of existing markers.

### Limitations

Short follow-up limited robustness of analysis with only seven recurrences and could not analyze for overall survival. As mentioned in Methods, cases of small sizes were excluded, which may have caused selection bias.

Immunostaining may have been affected by pre-analytical conditions and antigen preservation. Although MAI has been known to be relatively unaffected by fixation conditions, PHH3 and Ki67, both immunohistochemical stains, would be affected by fixation conditions.

Reproducibility study based on five raters was limited as these were pathologists from the same institution with similar training. Therefore, our reproducibility study may not fully represent the general cohort of pathologists. In addition, sample size of five rater inter-rater agreement was limited to 30 cases; evaluation under the microscope takes substantial time and effort while the generally accepted number which allows sufficient weight in analysis in inter-rater agreement is as few as 30 cases.

For direct comparison with Ki67 for expression qualities, clinicopathologic characteristics, including survival analysis, PHH3 was evaluated for the same fields previously selected by Ki67. Therefore, our results of PHH3 having strong significance in survival implies that assessment of PHH3 requires an initial selection of scoring fields by Ki67 to predict prognosis. For these reasons, though PHH3 proved to be an independent prognostic variable, possibly most important amongst conventional clinicopathologic markers, its independent application in breast cancer diagnostics merits further study. For now, we advocate the routine use of PHH3 in breast cancer diagnostics in conjunction with Ki67 to improve prediction of disease-free survival.

## MATERIALS AND METHODS

### Patient selection and clinicopathologic analysis

This retrospective study was approved by the Institutional Review Board (IRB) of Yonsei University Severance Hospital. 218 surgical tissues from 216 donor patients who had invasive ductal cancer, not otherwise specified, diagnosed and surgically resected at Severance Hospital from January 2012 to December 2013 were analyzed. All tissues were fixed in 10% buffered formalin for 48 hours then embedded in paraffin. All archival H&E-stained slides from all cases were reviewed by a breast pathologist (Koo JS). Cases treated with pre-operative chemotherapy or radiotherapy was excluded. Also, cases with tumor size smaller than 0.5cm were excluded, as considered too small to generate four HPFs for evaluation. Included clinical parameters were patient age at initial diagnosis, tumor stage, nodal metastasis, tumor recurrence and deaths. Histological grade was assessed by the Nottingham grading system [33].

### Immunohistochemistry (IHC)

Antibodies used for IHC are listed in Supplementary Table 1. All IHC was performed with formalin-fixed, paraffin-embedded tissue sections. Briefly, 5-μm-thick sections were obtained with a microtome, transferred onto adhesive slides, and dried at 62°C for 30 minutes.

Using the Discovery XT automated immunohistochemistry stainer (Ventana Medical Systems, Inc., Tucson, AZ, USA), slides were stained as the following procedure. Detection was done using the Ventana DAB Map Kit (Ventana Medical Systems).

Tissue sections were deparaffinized using EZ Prep solution. CC1 standard^®^ (pH 8.4 buffer containing Tris/Borate/EDTA) was used for antigen retrieval. Inhibitor D^®^ (3% H2O2, Endogenous peroxidase) was blocked for 4 min at 37°C temperature. Slides were incubated with primary antibodies (PHH3, Polyclonal, 1:100, Cell Marque, Rocklin, CA, USA; anti-Ki67 antibodies, clone MIB-1, DAKO, Glostrup, Denmark) for 40 min at 37°C, and a secondary antibody of biotinylated anti-mouse immunoglobulin for 20 min at 37°C. Slides were incubated in SA-HRP D^®^ (peroxidase-labeled streptavidin using a labeled streptavidin biotin kit) for 16 min, at 37°C and then 3,3′-diaminobenzidine chromogen combined H2O2 substrate for 8 min followed by Harris hematoxylin and bluing reagent counterstain at 37°C for 4 minutes. Reaction buffer (pH 7.6 Tris buffer) was used as washing solution. Tonsilar tissue was used for both positive and negative controls.

### IHC interpretations

All IHC markers were assessed by light microscopy. A cut-off value of 1% or more positively stained nuclei was used to define ER and PR positivity [34]. HER-2 staining was analyzed according to the American Society of Clinical Oncology (ASCO)/College of American Pathologists (CAP) guidelines using the following categories: 0 = no immunostaining, 1+ = weak incomplete membranous staining in less than 10% of tumor cells, 2+ = complete membranous staining, either uniform or weak in at least 10% of tumor cells, and 3+ = uniform intense membranous staining in at least 30% of tumor cells [35]. HER-2 was considered positive when strong (3+) membranous staining was observed, whereas cases with 0 to 1+ scores were regarded as negative. Cases showing 2+ HER-2 expression were further evaluated for HER-2 amplification by fluorescence *in situ* hybridization (FISH).

### Tumor phenotype classification

Breast cancer phenotypes classified according to IHC results for ER, PR, HER-2, Ki67, and FISH results for HER-2 as follows [36]. Luminal A, ER or/and PR positive, HER-2 negative and Ki67 labeling index (LI) < 14%; luminal B (HER-2 negative), HER-2 negative ER or/and PR positive, HER-2 negative and Ki67 LI ≥ 14%; luminal B(HER-2 positive), ER or/and PR positive and HER-2 overexpressed or/and amplified; HER-2 enriched, ER and PR negative and HER-2 overexpressed or/and amplified; and TNBC, ER, PR, and HER-2 negative.

### Selection of fields

Ki67 was examined first to select appropriate fields. Four HPFs (objective 40x) that best represented the overall tumor were selected from the invasive front, in the manner recommended by the International Ki67 in Breast Cancer Working Group [20]. When hot spots were present, these were included in the overall average score. The same four fields were appropriately marked for PHH3 (Supplementary Figure 1). Each field of examination was photographed to assure consistency of the field of examination at a given time.

Tumor cells were counted manually using the counter application of publicly available image analysis program, Image J.

### Scoring Ki67 and PHH3

In scoring Ki67, tumor cells were considered positive for only strongly stained nuclei, which were clearly above the background level, in keeping with previous studies [3]. Intact nuclei with fine granular staining of PHH3 were not counted as these cells were not in mitosis, but only strongly stained cells with mitogenic morphology was counted [37]. A percentage score was obtained by dividing the number of positively stained cells by the total number of cells. The final percentage score was the average of four fields.

### Inter-rater reproducibility study of five raters

Five pathologists working in the same institute participated in the inter-rater reproducibility study of Ki67 and PHH3 for 30 cases. H&E slides of the IHC stained slides were jointly provided for reference before scoring each case. Each participant’s ratings were made independently without knowledge of each other’s ratings as each test was conducted at different times without one knowing who was participating in this study. The first author gave same instructions for each participant and monitored the progress. There were no chances of discussion amongst participants. All are qualified pathologists who are reporting actively on cancer biomarker results.

### Statistical analysis

Data were analyzed using SPSS for Windows, Version 21.0 (SPSS Inc., Chicago, IL, USA), SAS, Version 9.4 (SAS Institute Inc., SAS Campus Drive, Cary, North Carolina 27513, USA), R statistics, Version 3.2.2 (The R Foundation for Statistical Computing, Vienna, Austria). Ki67 and PHH3 scores were calculated as categorical variables. For determination of statistical significance Chi square cross analysis was used for dichotomous variables. Correlation was analyzed by linear regression analysis. For inter-rater agreement, ICC and Cohen’s κ statistics were used where ICC was used to assess inter-rater reliability for numeric variables while Cohen’s κ was used to assess inter-rater reliability for categorical variables. ICC was compared for PHH3 and Ki67 according to their corresponding different scoring methods using the same cases, using comparison test of dependent ICC (Supplementary Figure 2) [38]. Cut-off determination by Contal and O’Quigely’s method was performed on SAS. The AIC and Harrell’s C-index was calculated for Ki67 and PHH3 each, on R statistics. Statistical significance was set to *P* < 0.05. Kaplan–Meier survival curves and log-rank statistics were used to evaluate time to tumor recurrence. Cox proportional hazards model was used for univariate regression analysis.

## SUPPLEMENTARY MATERIALS FIGURES AND TABLES




