# Supplementary material for: The value of phosphohistone H3 as a proliferation marker for evaluating invasive breast cancers: A comparative study with Ki67
Source: Oncotarget. 2017 May 10;8(39):65064–76. doi: 10.18632/oncotarget.17775 (PMC5630312; doi:10.18632/oncotarget.17775)
Supplement: Supplementary file 1 [file oncotarget-08-65064-s001.pdf]

The value of phosphohistone H3 as a proliferation marker for evaluating invasive breast cancers: A comparative study with Ki67

Supplementary Materials

Supplementary Table 1: Antibody sources, clones, and dilutions

Antibody	Clone	Dilution	Vendor
ER	SP1	1:100	Thermo Scientific, CA, USA
PR	PgR	1:50	DAKO, Glostrup, Denmark
HER-2	Polyclonal	1:1500	DAKO, Glostrup, Denmark
Ki-67	MIB1	1:100	DAKO, Glostrup, Denmark
PHH3	Polyclonal	1:100	Cell Marque, Rocklin, CA, USA

ER, estrogen receptor; PR, progesterone receptor

Supplementary Table 2: Ki67 and PHH3 cut-off value calculated by Contal and O'Quigley's method (run on SAS). See Supplementary_Table_2

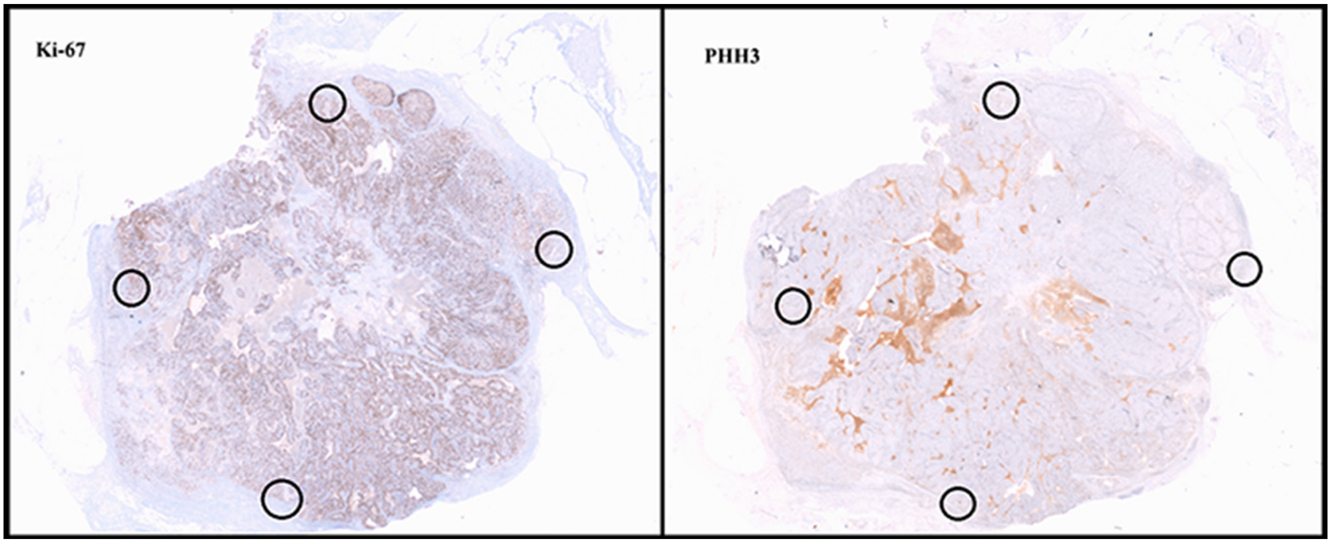

Supplementary Figure 1: Ki67 was examined first to select appropriate fields. After scanning the entire tumor area at low power, four HPFs (objective 40×) that best represented the overall tumor were selected from the invasive front of the tumor, including hot spots. The same four fields were appropriately marked for PHH3. Each field of examination was then photographed for scoring.

Let x_i, y_i i^{th} subject of variable x and y. Variance is denoted by V(・)

$$H_0 : ICC_1 = ICC_2 \text{ vs. } H_1 : ICC_1 \neq ICC_2$$

$$Z = \frac{Z_1 - Z_2}{\sqrt{V(Z_1) + V(Z_2) - 2\,\mathrm{cov}(Z_1, Z_2)}}$$

$$\text{where, } \mathrm{cov}\left(Z_1, Z_2\right) = \frac{k_1 k_2 \rho_{12}^2}{2n\{1 + \left(k_1 - 1\right) ICC_1 \left\{1 + (k_2 - 1\right\} ICC_2\right\}}$$

$$\rho_{12} = \frac{cov\left(\bar{x}_i, \bar{y}_i\right)}{\sqrt{\left\{2V\left(\bar{x}_i\right) - cov\left(\bar{x}_i, \bar{y}_i\right)\right\}\{2V\left(\bar{y}_i\right) - cov\left(\bar{x}_i, \bar{y}_i\right)\}}}$$

$$Z_i = \frac{1}{2} ln\left\{1 + \frac{\left(k_i - 1\right) ICC_i}{1 - ICC_i}\right\}$$

ICC1 = 0.712, n1 = 30, k1 = 5
ICC2 = 0.904, n2 = 30, k2 = 5

Supplementary Figure 2: Comparison of dependent ICC.
