# Supplementary material for: The value of phosphohistone H3 as a proliferation marker for evaluating invasive breast cancers: A comparative study with Ki67
Source: Oncotarget. 2017 May 10;8(39):65064–76. doi: 10.18632/oncotarget.17775 (PMC5630312; doi:10.18632/oncotarget.17775)
Supplement: Supplementary file 2 [file oncotarget-08-65064-s002.docx]

**Supplementary Table S2.** Ki67 and PHH3 cut-off value calculated by Contal and O’Quigley’s method (run on SAS)

| **Obs** | **Cutpoint** | **ki67** | **sk** | **ABS(sk)** | **Q statistics** | **P-value** | **pick** |
| --- | --- | --- | --- | --- | --- | --- | --- |
| **1** | 1 | 0 | 0.00000 | 0.00000 | 0.00000 | P>0.30 |  |
| **2** | 2 | 1 | 0.03593 | 0.03593 | 0.01712 | P>0.30 |  |
| **3** | 3 | 2 | 0.16694 | 0.16694 | 0.07952 | P>0.30 |  |
| **4** | 4 | 3 | 0.20287 | 0.20287 | 0.09664 | P>0.30 |  |
| **5** | 5 | 4 | 0.44805 | 0.44805 | 0.21342 | P>0.30 |  |
| **6** | 6 | 5 | 0.62772 | 0.62772 | 0.29901 | P>0.30 |  |
| **7** | 7 | 6 | 0.77146 | 0.77146 | 0.36748 | P>0.30 |  |
| **8** | 8 | 7 | 0.84333 | 0.84333 | 0.40172 | P>0.30 |  |
| **9** | 9 | 8 | 0.87926 | 0.87926 | 0.41883 | P>0.30 |  |
| **10** | 10 | 9 | 1.01026 | 1.01026 | 0.48123 | P>0.30 |  |
| **11** | 11 | 10 | 1.18357 | 1.18357 | 0.56379 | P>0.30 |  |
| **12** | 12 | 11 | 0.54292 | 0.54292 | 0.25862 | P>0.30 |  |
| **13** | 13 | 12 | 0.80903 | 0.80903 | 0.38538 | P>0.30 |  |
| **14** | 14 | 13 | 0.96959 | 0.96959 | 0.46186 | P>0.30 |  |
| **15** | 15 | 14 | 1.13016 | 1.13016 | 0.53835 | P>0.30 |  |
| **16** | 16 | 15 | 1.19566 | 1.19566 | 0.56955 | P>0.30 |  |
| **17** | 17 | 16 | 0.45687 | 0.45687 | 0.21763 | P>0.30 |  |
| **18** | 18 | 17 | 0.79711 | 0.79711 | 0.37970 | P>0.30 |  |
| **19** | 19 | 18 | 1.00635 | 1.00635 | 0.47937 | P>0.30 |  |
| **20** | 20 | 19 | 1.14372 | 1.14372 | 0.54480 | P>0.30 |  |
| **21** | 21 | 20 | 0.26967 | 0.26967 | 0.12845 | P>0.30 |  |
| **22** | 22 | 21 | 0.41182 | 0.41182 | 0.19617 | P>0.30 |  |
| **23** | 23 | 22 | 0.54919 | 0.54919 | 0.26160 | P>0.30 |  |
| **24** | 24 | 23 | 0.71613 | 0.71613 | 0.34112 | P>0.30 |  |
| **25** | 25 | 24 | 0.87801 | 0.87801 | 0.41824 | P>0.30 |  |
| **26** | 26 | 25 | 1.08725 | 1.08725 | 0.51791 | P>0.30 |  |
| **27** | 27 | 26 | 1.18869 | 1.18869 | 0.56623 | P>0.30 |  |
| **28** | 28 | 27 | 1.28375 | 1.28375 | 0.61151 | P>0.30 |  |
| **29** | 29 | 28 | 1.40612 | 1.40612 | 0.66980 | P>0.30 |  |
| **30** | 30 | 29 | 1.57943 | 1.57943 | 0.75235 | P>0.30 |  |
| **31** | 31 | 30 | 0.77367 | 0.77367 | 0.36853 | P>0.30 |  |
| **32** | 32 | 31 | 0.87989 | 0.87989 | 0.41913 | P>0.30 |  |
| **33** | 33 | 32 | 0.94539 | 0.94539 | 0.45033 | P>0.30 |  |
| **34** | 34 | 33 | 1.05319 | 1.05319 | 0.50168 | P>0.30 |  |
| **35** | 35 | 34 | 1.18419 | 1.18419 | 0.56408 | P>0.30 |  |
| **36** | 36 | 35 | 1.25606 | 1.25606 | 0.59832 | P>0.30 |  |
| **37** | 37 | 36 | 1.39343 | 1.39343 | 0.66375 | P>0.30 |  |
| **38** | 38 | 37 | 1.49487 | 1.49487 | 0.71207 | P>0.30 |  |
| **39** | 39 | 38 | 1.56037 | 1.56037 | 0.74327 | P>0.30 |  |
| **40** | 40 | 39 | 1.62587 | 1.62587 | 0.77447 | P>0.30 |  |
| **41** | 41 | 40 | 1.66180 | 1.66180 | 0.79159 | P>0.30 |  |
| **42** | 42 | 41 | 1.69774 | 1.69774 | 0.80871 | P>0.30 |  |
| **43** | 43 | 42 | 1.86467 | 1.86467 | 0.88823 | P>0.30 |  |
| **44** | 44 | 43 | 1.90061 | 1.90061 | 0.90534 | P>0.30 | <==== |
| **45** | 45 | 44 | 0.93017 | 0.93017 | 0.44308 | P>0.30 |  |
| **46** | 46 | 46 | 0.96611 | 0.96611 | 0.46020 | P>0.30 |  |
| **47** | 47 | 47 | 1.00204 | 1.00204 | 0.47732 | P>0.30 |  |
| **48** | 48 | 48 | 1.03798 | 1.03798 | 0.49444 | P>0.30 |  |
| **49** | 49 | 49 | 0.09843 | 0.09843 | 0.04689 | P>0.30 |  |
| **50** | 50 | 50 | 0.13436 | 0.13436 | 0.06400 | P>0.30 |  |
| **51** | 51 | 51 | 0.19986 | 0.19986 | 0.09520 | P>0.30 |  |
| **52** | 52 | 53 | 0.23580 | 0.23580 | 0.11232 | P>0.30 |  |
| **53** | 53 | 57 | 0.27173 | 0.27173 | 0.12944 | P>0.30 |  |
| **54** | 54 | 58 | 0.34360 | 0.34360 | 0.16367 | P>0.30 |  |
| **55** | 55 | 59 | 0.37954 | 0.37954 | 0.18079 | P>0.30 |  |
| **56** | 56 | 61 | 0.45141 | 0.45141 | 0.21503 | P>0.30 |  |
| **57** | 57 | 62 | 0.48734 | 0.48734 | 0.23214 | P>0.30 |  |
| **58** | 58 | 65 | 0.52328 | 0.52328 | 0.24926 | P>0.30 |  |
| **59** | 59 | 66 | 0.55921 | 0.55921 | 0.26638 | P>0.30 |  |
| **60** | 60 | 69 | 0.56400 | 0.56400 | 0.26866 | P>0.30 |  |
| **61** | 61 | 70 | 0.59993 | 0.59993 | 0.28577 | P>0.30 |  |
| **62** | 62 | 71 | 0.63587 | 0.63587 | 0.30289 | P>0.30 |  |
| **63** | 63 | 72 | 0.70774 | 0.70774 | 0.33713 | P>0.30 |  |
| **64** | 64 | 73 | 0.74367 | 0.74367 | 0.35424 | P>0.30 |  |
| **65** | 65 | 78 | 0.77961 | 0.77961 | 0.37136 | P>0.30 |  |
| **66** | 66 | 79 | 0.81554 | 0.81554 | 0.38848 | P>0.30 |  |
| **67** | 67 | 83 | -0.14374 | 0.14374 | 0.06847 | P>0.30 |  |
| **68** | 68 | 84 | -0.10780 | 0.10780 | 0.05135 | P>0.30 |  |
| **69** | 69 | 85 | -0.07187 | 0.07187 | 0.03423 | P>0.30 |  |
| **70** | 70 | 89 | -0.03593 | 0.03593 | 0.01712 | P>0.30 |  |

| **Obs** | **Cutpoint** | **phh3** | **sk** | **ABS(sk)** | **Q statistics** | **P-value** | **pick** |
| --- | --- | --- | --- | --- | --- | --- | --- |
| **1** | 1 | 0.00 | 0.00000 | 0.00000 | 0.00000 | P>0.30 |  |
| **2** | 2 | 0.03 | 2.39757 | 2.39757 | 1.14207 | 0.1473 |  |
| **3** | 3 | 0.04 | 2.42713 | 2.42713 | 1.15615 | 0.138 |  |
| **4** | 4 | 0.05 | 2.46307 | 2.46307 | 1.17327 | 0.1275 |  |
| **5** | 5 | 0.06 | 2.53494 | 2.53494 | 1.20750 | 0.1083 |  |
| **6** | 6 | 0.07 | 1.61159 | 1.61159 | 0.76767 | P>0.30 |  |
| **7** | 7 | 0.09 | 1.61159 | 1.61159 | 0.76767 | P>0.30 |  |
| **8** | 8 | 0.10 | 1.74259 | 1.74259 | 0.83007 | P>0.30 |  |
| **9** | 9 | 0.11 | 1.84403 | 1.84403 | 0.87839 | P>0.30 |  |
| **10** | 10 | 0.12 | 1.95183 | 1.95183 | 0.92975 | P>0.30 |  |
| **11** | 11 | 0.13 | 2.08920 | 2.08920 | 0.99518 | P>0.30 |  |
| **12** | 12 | 0.14 | 2.12514 | 2.12514 | 1.01230 | 0.2576 |  |
| **13** | 13 | 0.16 | 2.41103 | 2.41103 | 1.14848 | 0.143 |  |
| **14** | 14 | 0.18 | 2.44697 | 2.44697 | 1.16560 | 0.1321 |  |
| **15** | 15 | 0.19 | 2.48290 | 2.48290 | 1.18272 | 0.1219 |  |
| **16** | 16 | 0.20 | 2.55477 | 2.55477 | 1.21695 | 0.1034 |  |
| **17** | 17 | 0.21 | 2.59071 | 2.59071 | 1.23407 | 0.0951 |  |
| **18** | 18 | 0.22 | 2.65621 | 2.65621 | 1.26527 | 0.0814 |  |
| **19** | 19 | 0.23 | 2.72171 | 2.72171 | 1.29647 | 0.0694 |  |
| **20** | 20 | 0.24 | 2.79358 | 2.79358 | 1.33071 | 0.0579 |  |
| **21** | 21 | 0.26 | 2.86545 | 2.86545 | 1.36494 | 0.0482 |  |
| **22** | 22 | 0.27 | 2.90138 | 2.90138 | 1.38206 | 0.0438 |  |
| **23** | 23 | 0.28 | 3.03875 | 3.03875 | 1.44749 | 0.0303 |  |
| **24** | 24 | 0.29 | 3.07469 | 3.07469 | 1.46461 | 0.0274 |  |
| **25** | 25 | 0.30 | 3.14656 | 3.14656 | 1.49885 | 0.0224 | <==== |
| **26** | 26 | 0.31 | 2.18249 | 2.18249 | 1.03962 | 0.2303 |  |
| **27** | 27 | 0.33 | 2.21843 | 2.21843 | 1.05674 | 0.2143 |  |
| **28** | 28 | 0.34 | 2.25436 | 2.25436 | 1.07385 | 0.1993 |  |
| **29** | 29 | 0.35 | 1.33589 | 1.33589 | 0.63635 | P>0.30 |  |
| **30** | 30 | 0.36 | 1.40776 | 1.40776 | 0.67058 | P>0.30 |  |
| **31** | 31 | 0.37 | 1.45189 | 1.45189 | 0.69160 | P>0.30 |  |
| **32** | 32 | 0.39 | 1.55970 | 1.55970 | 0.74295 | P>0.30 |  |
| **33** | 33 | 0.40 | 1.62520 | 1.62520 | 0.77415 | P>0.30 |  |
| **34** | 34 | 0.41 | 1.69707 | 1.69707 | 0.80839 | P>0.30 |  |
| **35** | 35 | 0.44 | 1.76894 | 1.76894 | 0.84262 | P>0.30 |  |
| **36** | 36 | 0.46 | 1.80487 | 1.80487 | 0.85974 | P>0.30 |  |
| **37** | 37 | 0.47 | 1.84081 | 1.84081 | 0.87686 | P>0.30 |  |
| **38** | 38 | 0.48 | 1.87674 | 1.87674 | 0.89398 | P>0.30 |  |
| **39** | 39 | 0.49 | 1.91268 | 1.91268 | 0.91109 | P>0.30 |  |
| **40** | 40 | 0.50 | 1.94861 | 1.94861 | 0.92821 | P>0.30 |  |
| **41** | 41 | 0.51 | 2.01411 | 2.01411 | 0.95941 | P>0.30 |  |
| **42** | 42 | 0.52 | 2.05005 | 2.05005 | 0.97653 | P>0.30 |  |
| **43** | 43 | 0.54 | 2.11555 | 2.11555 | 1.00773 | 0.2624 |  |
| **44** | 44 | 0.55 | 2.12033 | 2.12033 | 1.01001 | 0.26 |  |
| **45** | 45 | 0.58 | 2.18583 | 2.18583 | 1.04121 | 0.2288 |  |
| **46** | 46 | 0.59 | 2.22177 | 2.22177 | 1.05833 | 0.2129 |  |
| **47** | 47 | 0.60 | 2.25770 | 2.25770 | 1.07544 | 0.1979 |  |
| **48** | 48 | 0.61 | 2.25770 | 2.25770 | 1.07544 | 0.1979 |  |
| **49** | 49 | 0.62 | 2.29364 | 2.29364 | 1.09256 | 0.1837 |  |
| **50** | 50 | 0.64 | 2.32957 | 2.32957 | 1.10968 | 0.1704 |  |
| **51** | 51 | 0.66 | 2.39507 | 2.39507 | 1.14088 | 0.1481 |  |
| **52** | 52 | 0.67 | 2.46694 | 2.46694 | 1.17511 | 0.1264 |  |
| **53** | 53 | 0.73 | 1.52739 | 1.52739 | 0.72757 | P>0.30 |  |
| **54** | 54 | 0.74 | 1.56333 | 1.56333 | 0.74468 | P>0.30 |  |
| **55** | 55 | 0.75 | 0.58784 | 0.58784 | 0.28002 | P>0.30 |  |
| **56** | 56 | 0.76 | 0.65971 | 0.65971 | 0.31425 | P>0.30 |  |
| **57** | 57 | 0.78 | 0.69565 | 0.69565 | 0.33137 | P>0.30 |  |
| **58** | 58 | 0.80 | 0.73158 | 0.73158 | 0.34848 | P>0.30 |  |
| **59** | 59 | 0.81 | -0.13742 | 0.13742 | 0.06546 | P>0.30 |  |
| **60** | 60 | 0.82 | -0.10148 | 0.10148 | 0.04834 | P>0.30 |  |
| **61** | 61 | 0.84 | -0.06555 | 0.06555 | 0.03122 | P>0.30 |  |
| **62** | 62 | 0.86 | -0.02961 | 0.02961 | 0.01411 | P>0.30 |  |
| **63** | 63 | 0.87 | 0.04226 | 0.04226 | 0.02013 | P>0.30 |  |
| **64** | 64 | 0.91 | 0.07819 | 0.07819 | 0.03725 | P>0.30 |  |
| **65** | 65 | 0.95 | 0.15006 | 0.15006 | 0.07148 | P>0.30 |  |
| **66** | 66 | 1.04 | 0.17963 | 0.17963 | 0.08556 | P>0.30 |  |
| **67** | 67 | 1.05 | 0.21556 | 0.21556 | 0.10268 | P>0.30 |  |
| **68** | 68 | 1.07 | 0.25150 | 0.25150 | 0.11980 | P>0.30 |  |
| **69** | 69 | 1.09 | 0.28106 | 0.28106 | 0.13388 | P>0.30 |  |
| **70** | 70 | 1.10 | 0.31700 | 0.31700 | 0.15100 | P>0.30 |  |
| **71** | 71 | 1.18 | -0.66844 | 0.66844 | 0.31841 | P>0.30 |  |
| **72** | 72 | 1.22 | -0.63250 | 0.63250 | 0.30129 | P>0.30 |  |
| **73** | 73 | 1.23 | -0.59657 | 0.59657 | 0.28417 | P>0.30 |  |
| **74** | 74 | 1.25 | -0.56063 | 0.56063 | 0.26706 | P>0.30 |  |
| **75** | 75 | 1.29 | -0.55585 | 0.55585 | 0.26478 | P>0.30 |  |
| **76** | 76 | 1.36 | -0.51991 | 0.51991 | 0.24766 | P>0.30 |  |
| **77** | 77 | 1.37 | -0.49035 | 0.49035 | 0.23358 | P>0.30 |  |
| **78** | 78 | 1.40 | -0.46078 | 0.46078 | 0.21949 | P>0.30 |  |
| **79** | 79 | 1.42 | -0.42485 | 0.42485 | 0.20237 | P>0.30 |  |
| **80** | 80 | 1.51 | -0.38891 | 0.38891 | 0.18526 | P>0.30 |  |
| **81** | 81 | 1.56 | -0.35298 | 0.35298 | 0.16814 | P>0.30 |  |
| **82** | 82 | 1.58 | -0.31704 | 0.31704 | 0.15102 | P>0.30 |  |
| **83** | 83 | 1.77 | -0.28111 | 0.28111 | 0.13391 | P>0.30 |  |
| **84** | 84 | 1.78 | -0.24517 | 0.24517 | 0.11679 | P>0.30 |  |
| **85** | 85 | 1.82 | -0.20924 | 0.20924 | 0.09967 | P>0.30 |  |
| **86** | 86 | 1.88 | -0.17330 | 0.17330 | 0.08255 | P>0.30 |  |
| **87** | 87 | 1.89 | -0.10780 | 0.10780 | 0.05135 | P>0.30 |  |
| **88** | 88 | 2.15 | -0.07187 | 0.07187 | 0.03423 | P>0.30 |  |
| **89** | 89 | 2.22 | -0.03593 | 0.03593 | 0.01712 | P>0.30 |  |
